# Upregulation of sodium taurocholate cotransporter polypeptide during hepatogenic differentiation of umbilical cord matrix mesenchymal stem cells facilitates hepatitis B entry

**DOI:** 10.1186/s13287-017-0656-5

**Published:** 2017-09-29

**Authors:** Camillo Sargiacomo, Hoda El-Kehdy, Kai Dallmeier, Joery de Kock, Clara Hernandez-Kelly, Vera Rogiers, Arturo Ortega, Johan Neyts, Etienne Sokal, Mustapha Najimi

**Affiliations:** 1 Institute of Experimental and Clinical Research (IREC), Laboratory of Pediatric Hepatology & Cell Therapy, Université Catholique de Louvain, Avenue Mounier, 52, 1200 Brussels, Belgium; 2 Rega Institute for Medical Research, Department of Microbiology & Immunology, University of Leuven (KU Leuven), Leuven, Belgium; 3 Faculty of Medicine and Pharmacy, Department of Toxicology, Dermato-Cosmetology and Pharmacognosy, Vrije Universiteit Brussel, Brussels, Belgium; 4 Centro de Investigación y Estudios Avanzados del Instituto Politécnico Nacional (CINVESTAV-IPN),Departamento de Genética y Biología Molecular, México D.F, Mexico

**Keywords:** D-UCMSCs, Hepatogenic differentiation, Dexamethasone, NTCP modulation, HBV in vitro infection model

## Abstract

**Background:**

Hepatitis B virus (HBV) carriers worldwide number approximately 240 million people and around 780,000 people die every year from HBV infection. HBV entry and uptake are functionally linked to the presence of the human sodium-taurocholate cotransporting peptide (hNTCP) receptor. Recently, our group demonstrated that human umbilical cord matrix stem cells (UCMSCs) become susceptible to HBV after in-vitro hepatogenic differentiation (D-UCMSCs).

**Methods:**

In the present study, we examined the involvement of hNTCP in governing D-UCMSC susceptibility to HBV infection by characterizing the modulation of this transporter expression during hepatogenic differentiation and by appreciating the inhibition of its activity on infection efficacy.

**Results:**

We show here that in-vitro hepatogenic differentiation upregulated hNTCP mRNA and protein expression as well as its activity in D-UCMSCs. Pre-treatment of D-UCMSCs with taurocholate, a specific NTCP substrate, blocked their infection by HBV which supports the crucial involvement of this transporter in the early steps of the virus entry.

**Conclusion:**

Altogether, our data support the usefulness of D-UCMSCs as a unique human and non-transformed in-vitro model to study the early stages of HBV infection thanks to its ability to endogenously regulate the expression of hNTCP.

**Electronic supplementary material:**

The online version of this article (doi:10.1186/s13287-017-0656-5) contains supplementary material, which is available to authorized users.

## Background

Human hepatitis B virus (HBV) is a human pathogen with a restricted host range and a high specificity for liver cells. HBV infection affects 240 million people worldwide, and its transmission principally occurs during the neonatal period and early infancy [1]. Prophylactic vaccination against HBV has decreased the rate of chronic carriers by 70–90%. However, 10–30% of neonates do not respond to the vaccine and have an elevated risk of developing hepatocellular carcinoma later in adulthood [1]. Currently, there are two common anti-HBV therapies available: 1) nucleos(t)ide analogs that inhibit viral replication; and 2) conventional interferon (IFN)-alpha therapies that stimulate the immune system and indirectly block viral protein translation [2].

However, both therapies fail to eradicate infection as they are only blocking the viral genome replication, and neither eliminate the HBV covalently closed circular DNA (cccDNA) that serves as a long-lived episomal transcriptional template for HBV replication and reservoir for viral persistence, nor do they prevent entry and thus (re)infection of naive hepatocytes [3]. An alternative strategy would be the blockade of the viral entry process since, by acting on the cellular host components, it may avoid the development of drug-resistant viruses and, most importantly, it stops the process of re-infection such as after liver transplantation [4]. The lack of a fully suitable and easily available HBV in-vitro infection model hampers in-depth investigations of the HBV molecular entry mechanisms.

A few infection models have been developed and all of them present common suboptimal features [5]. Only primary human hepatocytes (PHHs) and hepatocytes of *Tupaia belangeri* (PTH) are susceptible to HBV infection in vitro, but their rapid de-differentiation in culture and low resistance to cryopreservation are strong limitations for regular availability. Alternatively, HepaRG cells are the only established cell line susceptible to HBV entry and replication after dimethyl sulfoxide (DMSO)-induced differentiation/infection [6, 7]. With the recent discovery of the sodium-taurocholate cotransporting peptide (NTCP) as the HBV/HDV receptor [8], new NTCP-based culture systems have been developed to study HBV entry mechanisms [9]. Among these, HepG2 clone lines overexpressing NTCP have been successfully generated and can allow 70% of infection efficiency by using 2.5% DMSO [7]. However, both HepG2-NTCP and HepaRG cells present chromosomal abnormalities besides the requirement of DMSO for efficient infection [5, 7].

Thanks to their self-renewal and multilineage differentiation potential, mesenchymal stem cells (MSCs) may be considered a valid alternative source for studying virus-cell interactions. The Wharton’s jelly of the umbilical cord (UC) is a mucoid connective tissue [10] from which MSC (UCMSCs) can be easily and reproducibly isolated. These cells are able to significantly differentiate at the morphological, genetic, and functional levels into hepatocyte-like cells (D-UCMSCs) [11]. We have previously shown that D-UCMSCs allow HBV attachment and entry under polyethylene (PEG) and DMSO-free conditions, but not in their undifferentiated naive state [12]. These findings suggest that the infection process in D-UCMSCs is most probably mediated by an induced cell surface determinant of hepatocytic lineage.

In the present study, we examined the susceptibility of D-UCMSCs to HBV infection in relation to a functional NTCP expression during hepatogenic differentiation. We prove that D-UCMSCs displayed an upregulated NTCP expression at both mRNA and protein levels, as well as a significant increase of its uptake activity. We also found that increased NTCP expression occurred at the last maturation step of a three-step differentiation protocol and was dexamethasone (Dexa)-dependent, as it could be inhibited by the anti-glucocorticoid antagonist RU486. Finally, we prove that HBV entry in D-UCMSCs is NTCP-mediated since pre-treatment of D-UCMSCs with taurocholate, a specific NTCP substrate and competitive inhibitor of HBV entry, blocked HBV infection. These data consolidate our previous finding regarding the susceptibility of D-UCMSCs for HBV infection [12] and highlight the usefulness of such in-vitro cell model to investigate early stages of viral transmission by modulating NTCP expression.

## Methods

### Isolation and culture of UCMSCs

UCMSCs were isolated from the umbilical cord Wharton’s Jelly by using an optimized isolation protocol as compared to the one we previously documented [11]. These changes have significantly enhanced the yield of recovered cells per weight of material as well as their quality, which has improved the standardization of cell plating density and the emergence period. Briefly, umbilical cords were collected in a specific conservation solution (HBSS without Ca^2+^ and Mg^2+^, 10% Fungizone, 1% penicillin/streptomycin (P/S); Life Technologies) at 4 °C and processed within 24 h. After extensive washing with phosphate-buffered saline (PBS) supplemented with 1% P/S, veins and arteries were removed; the remaining tissue was minced and incubated with digestion solution (HBSS (Life Technologies), calcium chloride 10 mM (Sigma), collagenase D (0.2% (w/v); Roche) and hyaluronidase (100 U/ml; Roche)) for 90 min at 37 °C with shaking at 250 rpm. After centrifugation, cell counting and viability were evaluated prior to plating. Cells were suspended in expansion medium (Dulbecco’s modified Eagle medium (DMEM; 1 g/L glucose (Life Technologies), 20% fetal bovine serum (Life Technologies), and 1% P/S (Life Technologies)) and seeded at 10,000 cells/cm^2^ on plastic tissue culture flasks (Greiner Bio-One BVBA/SPRL, Belgium) at 37 °C in humidified atmosphere (5% CO_2_). Twenty-four hours later the medium was changed to remove debris and non-adherent cells, whereas further medium changes were performed twice a week. After two sequential passages, well-characterized UCMSCs were cryopreserved until use. In the current study, UCMSCs were used between passage 4 and 10.

### In vitro hepatogenic differentiation

Differentiation studies were conducted on UCMSCs that had been seeded at 10,000 cells/cm^2^ on collagen I-coated flasks (BD). After reaching 90% confluence, the expansion medium was removed, cells were washed once with sterile PBS, and hepatogenic differentiation was started by switching to Iscove’s modified Dulbecco’s medium (IMDM; Life Technologies) serum-free medium in which specific growth factors/cytokines (Perpotech EC Ltd.) were added as a sequential multi-step protocol. Step 1 (20 ng/mL epidermal-growth factor (EGF) and 10 ng/mL basic fibroblast growth factor (bFGF)) lasted for 2 days. Then, Step 2 (20 ng/mL bFGF, 10 ng/mL hepatocyte growth factor (HGF), insulin-selenium-transferrin (ITS; Life Technologies) and 0.61 g/L nicotinamide (Sigma)) lasted for 9 days including three medium changes. Subsequently, Step 3 (20 ng/mL oncostatin M (OSM), 20 ng/mL HGF, 1% ITS, 0.61 g/L nicotinamide and 10^–6^ M Dexa (Sigma)) lasted for another 9 days including three medium changes. Recovered differentiated cells were analyzed at the expression and functional levels to evaluate the quality of the acquired hepatogenic differentiation. Once the hepatogenic differentiation protocol was completed, D-UCMSCs were incubated in a maintenance medium (IMDM supplemented with 10^–6^ M Dexa and 20 ng/mL HGF) in order to sustain the acquired hepatic features. Maintenance medium was also used during the HBV infection studies.

### HBV source and in-vitro infection of D-UCMSCs

HBV batch production was conducted in collaboration with the Rega Institute. The viral source was produced in vitro using the HepAD38 cell line containing HBV (genotype D, subtype *ayw*). Viral stocks were quantified and concentrated at 10^7^–10^8^ HBV/mL from HepAD38 conditioned medium, as previously reported by our group [12]. D-UCMSC infection was accomplished by incubating the virus for 24 h at 37 °C. Viral replication was monitored for 4 days post-infection every 24 h. Infection efficiency was established using different viral loads (10^5^, 10^4^, and 10^3^ multiplicities of infections (MOIs)). MOIs of 2500 were used as a standard infection inoculum in the study.

### Quantitative reverse-transcription polymerase chain reaction (qPCR) analysis

RNA from both UCMSCs and D-UCMSCs was extracted with Tripure reagent (Roche) following the manufacturer’s instructions, and was quantified using a Nanodrop™ (Thermo Scientific, USA). Two micrograms of total RNA were reverse transcribed (RT) through a High Capacity cDNA Reverse Transcription Kit (Life Technologies). Various gene mRNAs were analyzed by TaqMan® gene expression assays using StepOnePlus (Thermo Fisher, USA) (Table 1). The delta delta comparative method was performed between UCMCSCs and D-UCMSCs by initially selecting from 32 candidate housekeeping genes, the TATA-Box binding protein (TBP) and the Processing of precursor 4 (POP4) based on qBASE PLUS software analysis (Biogazelle, Belgium).

**Table 1:** Primers used for differentiated umbilical cord mesenchymal stem cell gene expression analysis

Gene	Code	Supplier
NTCP	Hs00161820_m1	ThermoFisher
CYP3A4	Hs00604506_m1	ThermoFisher
CYP7A1	Hs00167982_m1	ThermoFisher
POP4	Hs99999910_m1	ThermoFisher
TBP	Hs00198357_m1	ThermoFisher

### Intracellular and extracellular HBV quantification analyses

Intracellular DNA and RNA were extracted from infected D-UCMSCs to analyze the HBV cccDNA and pregenomic (pg)RNA transcripts, respectively, as key markers of HBV infection. For HBV cccDNA analysis, DNA was extracted by Tris-Phenol and treated by RNase A to exclude the presence of contaminating RNA [13]. Samples were then quantified by Nanodrop and analyzed by the digital droplet qPCR method (ddqPCR). With this method, HBV cccDNA TaqMan assay [14] detection was conducted in parallel with RNase P assay (multiplex assay) to normalize the cccDNA copies per cell (RNase P). cccDNA TaqMan assay amplification products were analyzed on 2% agarose gel. Viral RNA extraction was conducted as described in the RT-qPCR section. HBV transcripts were detected using the RC01 assay (Thermo Fisher; ID: AIS07DM), as previously validated [12]. The RC01 assay amplifies a specific region common to both HBV pgRNA and preC major viral transcripts. Relative quantification analysis of HBV transcripts was normalized by using two reference genes as described above. Extracellular DNA extraction from infected D-USMSC conditioned medium was performed using the PureLink® Viral RNA/DNA Mini Kit (Life Technologies) according to the manufacturer’s protocol. Briefly, DNA was eluted in 25 μL of water, of which 5 μL was analyzed by qPCR to determine the amount of HBV relaxed circular (RC) DNA viral particles secreted by D-UCMSCs. Absolute quantification by the WHO International standards linear regression method allowed us to evaluate the copy number of secreted viral particles by D-UCMSCs. Primer and probe sequences are reported in Table 2.

**Table 2:** Primers used for hepatitis B virus quantification analysis and cell copy number

Sequence	Code/catalog number	Supplier
HBV RC DNA_F	CAGCACCATGCAACTTTTTCAC	Thermo Fisher
HBV RC DNA_R	ATCAATGTCCATGCCCCAAA	Thermo Fisher
HBV RC DNA_probe	TGTCCTACTGTTCAAGCC	Thermo Fisher
HBV cccDNA_F	CTCCCCGTCTGTGCCTTCT	Tibmolbiol
HBV cccDNA_R	GCCCCAAAGCCACCCAAG	Tibmolbiol
HBV cccDNA_probe	CGTCGCATGGARACCACCGTGAACGCC	Tibmolbiol
RNase P	4316831	Thermo Fisher

### CYP3A4 activity assay

The hepatogenic differentiation potential of UCMSCs was assessed by the Lytic CYP3A4 P450-Glo™ assay (Promega, Belgium) following the manufacturer’s instructions with slight modifications. To measure CYP3A4 activity, each reaction contained 0.1 pmol of luciferin-IPA substrate which was incubated with 200,000 cells/well in a 96-well white plate for 4 h at 37 °C. The luminescent signal was produced by adding luciferin detection reagent for an additional 20 min at room temperature and under gentle shaking. Plate reading was fixed at a 10-s acquisition time by a luminometer (VICTOR 3, PerkinElmer, USA). CYP3A4 activity is expressed as the relative light units (RLU) net signal, which is calculated by subtracting no-cell control values (substrate only) from test compound values (cell and substrate).

### Western blot analysis

Total protein lysates were obtained by dissolving cell pellets in RIPA buffer (50 mM Tris Base, pH 8.0, 150 mM NaCl, 1% Triton X-100 (Sigma), 0.5% deoxycholate (Sigma, Belgium), 0.1% SDS, supplemented with protein inhibitors cocktail without EDTA (Roche)). Protein samples were shortly sonicated and incubated for 30 min at 4 °C prior to sample clarification by centrifugation (15 min 17,000 rpm at 4 °C). Subsequently, sample supernatants were collected and total protein quantification was performed (BCA Quantification kit, Thermo Fisher). Ninety micrograms of total protein extracts were dissolved in loading buffer (100 mM Tris-HCl (pH 6.8), glycerol 2% (v/v), SDS 4% (w/v), DTT 200 mM, bromophenol blue 0.2%), denatured at 95 °C for 5 min, loaded onto a 10% Tris-glycine SDS-PAGE gel for protein separation, and transferred overnight at 4 °C onto PVDF membranes. Thereafter, membranes were incubated with 5% bovine serum albumin (BSA) blocking solution for 1 h at room temperature. Primary antibodies (Table 3) were incubated overnight at 4 °C; membranes were washed three times with PBS-Tween-20 (T) (1%; Sigma) and then incubated with fluorescently labeled secondary antibodies (Biotium) for 40 min at room temperature. Membranes were then washed (3× PBS-T) and fluorescence signals were detected by Li-cor scanner (Odyssey). Quantification analysis was performed by Image Studio Lite Software (Odyssey). For NTCP deglycosylation assay, 90 μg of total protein extracts were treated with PGNase F (New England Biolabs) for 2 h at 37 °C in RIPA buffer. A goat polyclonal anti-actin antibody (1:2000; sc-1616, Santa Cruz Biotechnology) served as the loading control

**Table 3:** Primary antibody references and working concentrations

Antigen	Supplier	Reference	Species	Type	Dilution
NTCP	Pr. Bruno Stieger	de Graaf et al., 2011 [17]	Rabbit	Polyclonal	1/1000
NTCP	Sigma	HPA042727	Rabbit	Polyclonal	1/1000
CYP3A4	Enzo Life Sciences	BML-CR3340-0025	Rabbit	Monoclonal	1/1000
GAPDH	Abcam	ab8245	Mouse	Monoclonal	1/10.000
Actin	Sigma	A2066	Rabbit	Polyclonal	1/1000

### Indocyanine green (ICG) uptake/release assay

ICG is a non-toxic tricarbocyanin dye exclusively taken up by hepatocytes through the LST1 (Liver-Specific organic Transporter-1). Naive and D-UCMSCs were incubated for 5 h with 1 mg/mL ICG (Sigma) at 37 °C. The uptake and the clearance of ICG were assessed on the same microscopic fields using a white light inverted microscope (Leica DM IL).

### Taurocholate uptake assay

After culture medium removal, cells were washed twice and incubated for 10 min with transport assay buffer at 37 °C (5.3 mM KCl, 1.1 mM KH_2_PO_4_, 0.8 mM MgSO_4_, 1.8 mM CaCl_2_, 11 mM d-glucose, 10 mM HEPES, and 136 mM NaCl (sodium-containing buffer)) [15]. To evaluate NTCP uptake activity, cells were incubated with pre-heated sodium-containing buffer supplemented with the radiolabeled [^3^H]-taurocholic acid substrate (1 μCi/ml; Perkin Elmer, Belgium). Different concentrations were applied (0 μM, 0.2 μM, 0.4 μM, 0.6 μM, 0.8 μM, and 1 μM) either in the presence or in the absence of sodium ions on both naive UCMSCs and D-UCMSCs. Uptake was stopped by adding ice-cold PBS containing 0.2% w/v BSA [16]. After a final washing step with ice-cold PBS, cells were lysed with mammalian protein extraction reagent (Pierce, Belgium) and the intracellular accumulation of the radiolabeled substrate was measured using liquid scintillation counting (Tri-Carb, Perkin Elmer, Belgium).

### Statistical analyses

Statistical analysis was performed with GraphPad PRISM 6 Software (GraphPad Software, USA). Mann-Whitney *U*, Wilcoxon signed-rank, one-sample two-tailed *t* test, and one-way analysis of variance (ANOVA) tests were used, as appropriate, and *p* < 0.05 was considered significant. Values are expressed as mean ± standard deviation (SD) or mean ± standard error of the mean (SEM).

## Results

### The effect of hepatogenic differentiation on UCMSCs

**Fig. 1:**
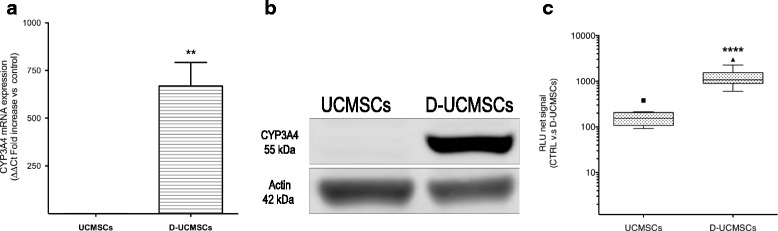
Hepatogenic differentiation of UCMSCs. **a** CYP3A4 mRNA expression is significantly induced in differentiated umbilical cord mesenchymal stem cells (*D-UCMSCs*). Values are expressed as fold increase (669 ± 71 SD; *n* = 3 vs. control UCMSCs (*n* = 3). Gene expression was normalized by POP4 and TBP housekeeping genes (*p* = 0.005; Mann-Whitney *U* test). **b** Upregulation of CYP3A4 protein expression in D-UCMSCs is demonstrated by Western blot; 90 μg of total protein extracts were loaded per well. Actin was used as the loading control. The blot is representative of three different donors tested. **c** CYP3A4 activity was also enhanced as demonstrated by using Luciferase assay in D-UCMSCs (1069 ± 157 SD, *n* = 16) when compared to UCMSCs (*CTRL*; 153 ± 32 SD, *n* = 8; *p* < 0.0001 by Mann-Whitney *U* test). Values are expressed as the relative light unit (*RLU*) net signal

All freshly isolated UCMSCs were characterized by flow cytometry using the MSC markers CD90, CD105, CD73, and CD45 (Additional file 1: Figure S1A) and subsequently cryopreserved as described in the Methods section. Hepatogenic differentiation was conducted on thawed UCMSCs with an optimized three-step protocol. Figure 1a shows that hepatogenic differentiation of UCMSCs significantly upregulates CYP3A4 mRNA expression in D-UCMSCs by at least 600 fold (*n* = 3) as compared to their naive counterparts (*t* test *p* = 0.005). Western blot analysis confirmed the increased CYP3A4 protein expression in D-UCMSCs. Indeed, a corresponding 55-kDa band was significantly induced in D-UCMSCs as compared to naive UCMSCs (Fig. 1b). Finally, to assess protein functionality, CYP3A4 activity was also assayed in D-UCMSCs. Figure 1c shows a significant increase in CYP3A4 activity in D-UCMSCs by at least 10 fold (*n* = 16) compared to naive UCMSCs (*n* = 8) (*t* test; *p* < 0.0001). These results indicate, along with other in-house specifications (Additional file 1: Figure S1A), that D-UCMSCs from the optimized isolation protocol acquired similar hepatocyte-like features as previously reported (Additional file 1: Figure S1B) [12].

### HBV infection of D-UCMSCs

**Fig. 2:**
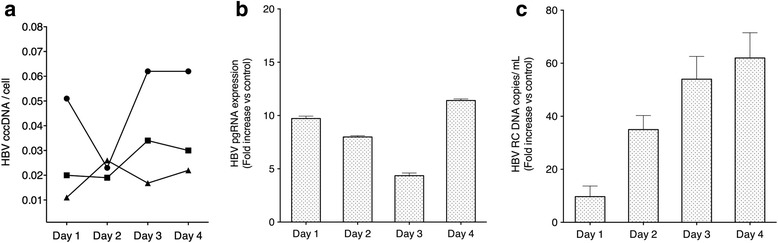
HBV infection kinetics in D-UCMSCs. **a** Hepatitis B virus (*HBV*) covalently closed circular DNA (*cccDNA*) kinetic: infected D-UCMSC samples were collected during 4 days post-infection and were analyzed by ddPCR multiplex assay. HBV cccDNA biogenesis was detectable in D-UCMSCs starting at day 1 up to day 4 post-infection (*n* = 3). The cell copy number was calculated by RNase P genome detection. Absolute values are expressed as HBV cccDNA copies per cell (donors 112, 114, and 116). **b** HBV pgRNA detection: viral transcripts (pregenomic (*pg*) and preC RNAs) were detected by qPCR during HBV infection kinetic in D-UCMSCs. Viral RNAs were detected starting at day 1 and expressed as fold-increase in time as compared to cells infected after 24 h after primary infection (donors 112 and 116). Relative quantification analysis of HBV pg/pc RNAs was normalized by using TBP and POP4 reference genes. **c** HBV relaxed circular (*RC*) DNA virion secretion: D-UCMSC infection kinetic studies show an increased production of HBV infectious particles over 4 days (*p* < 0.05). Absolute quantification of HBV RC DNA positive virions is expressed as fold change versus cells 24 hours after primary infection (donors 112 and 116)

Infection experiments were conducted over 4 days after incubating D-UCMSCs with HBV for 24 h at 37 °C. To assess the relative infectivity of HBV inocula, D-UCMSCs were infected with three viral titers (100, 1000, and 10,000 virions/cell determined as viral genome equivalents). As a result, we established 2500 HBV viral particles per cell as a standard infection protocol of D-UCMSCs for the current study (Additional file 1: Figure S2A). Soon after D-UCMSC infection, the presence of viral genome (cccDNA) became measurable by ddPCR. After validation of this sensitive method for DNA quantification (Additional file 1: Figure S2B), we accurately determined the absolute copy numbers of cccDNA per cell. Hence, Fig. 2a shows that cccDNA formation was detected over 4 days post-infection indicating its rapid formation in the nucleus of D-UCMSCs. Intracellular viral RNA transcript formation was monitored by RT-qPCR analysis of HBV pgRNA/preC transcripts. Figure 2b shows that viral replication in D-UCMSCs was successfully established and was sustained over 4 days post-infection. Finally, to confirm that infected D-UCMSCs support the entire HBV life cycle, production and secretion of RC DNA containing HBV virions was measured by absolute qPCR quantification. As described in Fig. 2c, D-UCMSCs produced detectable amounts of HBV virions and increased progeny production overtime as compared to day 1 post-infection.

### NTCP mRNA modulation in D-UCMSCs

**Fig. 3:**
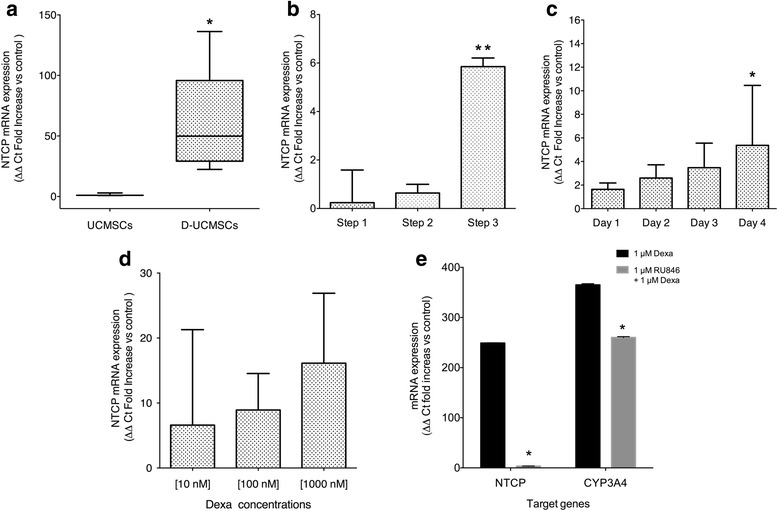
Characterization of NTCP mRNA expression in D-UCMSCs. **a** Sodium-taurocholate cotransporting peptide (*NTCP*) mRNA expression: hepatogenic differentiation induces NTCP mRNA modulation in differentiated umbilical cord mesenchymal stem cells (*D-UCMSCs*; 60.0 ± 44.5 SD, *n* = 5) as compared to UCMSCs (1.6 ± 1.2 SD, *n* = 3; *p* = 0.0357 by Mann-Whitney *U* test, two-tailed). The relative quantification method was normalized using TBP and POP4 reference genes. **b** NTCP expression at Step 3 of differentiation: the third step of hepatogenic differentiation induces NTCP mRNA expression (5.9 ± 0.2 SD) as compared to Step 2 (0.6 ± 0.2 SD) and Step 1 (0.2 ± 0.7 SD). Values are expressed as fold increase compared to UCMSCs (*p* = 0.0047 by unpaired *t* test, two-tailed; *n* = 3). **c** NTCP mRNA expression post-hepatogenic differentiation: maintenance medium containing dexamethasone (*Dexa*) 10^–6^ M and HGF 10 ng/ml was added at the end of the hepatogenic differentiation protocol. The NTCP mRNA expression kinetic was sustained and increased at day 4 post-differentiation (*p* = 0.05 by Mann-Whitney *U* test, one-tailed; *n* = 3). Values are expressed as ΔΔ Ct fold increase as compared to day 0 (end of differentiation). **d** The effect of Dexa on NTCP mRNA expression: D-UCMSCs were treated with Dexa (10 nM, 100 nM, and 1000 nM) at Step 3 of hepatogenic differentiation. NTCP mRNA levels were measured by RT-qPCR (*n* = 2). NTCP expression was increased in a dose-dependent manner by Dexa (6.5 ± 1.6 SD 10 nM; 8.4 ± 0.6 SD 100 nM; and 16.1 ± 1.2 SD 1000 nM). Values are expressed as ΔΔ Ct fold increase versus Step 2 (end of differentiation). **e** Inhibition of NTCP mRNA expression in D-UCMSCs treated by RU486. 1 μM RU486 was added at the end of hepatogenic differentiation for 4 days with Dexa 10^–6^ M. qPCR analysis revealed that NTCP mRNA expression was significantly reduced by treatment with 1 μM RU486 as well as CYP3A4 mRNA expression (*p* < 0.001 by multiple *t* test). Values expressed as relative quantity compared to UCMSCs

At the end of the hepatogenic differentiation process, D-UCMSCs were analyzed to test if NTCP expression was modulated. NTCP mRNA expression levels were initially measured by RT-qPCR. In Fig. 3a, we show that D-UCMSC expression of NTCP mRNA (60.0 ± 44.5 SD, *n* = 5) was significantly increased as compared to naive UCMSCs (1.6 ± 1.2 SD, *n* = 3; *p* = 0.0357 by Mann-Whitney *U* test). To evaluate the kinetics of NTCP mRNA upregulation, the end point of each of the three steps of the hepatogenic differentiation protocol was analyzed. Figure 3b shows that NTCP mRNA was significantly upregulated only at the end of the last step of the hepatogenic differentiation as compared to Step 1 (*p* = 0.0047; *t* test) and Step 2 (*p* = 0.0047; *t* test). Moreover, NTCP expression continued increasing (*p* < 0.001; *t* test) for at least 4 days after the end of the hepatogenic differentiation while only the maintenance medium containing Dexa and HGF was used (Fig. 3c). One of the main components of the maturation step that could modulate NTCP expression is Dexa. Consequently, NTCP mRNA levels were measured in D-UCMSCs on Dexa treatment with three different concentrations (10 nM, 100 nM, and 1000 nM). As shown in Fig. 3d, Dexa enhances NTCP mRNA expression in a dose-dependent (*p* = 0.009) and also in a time-dependent manner in D-UCMSCs (Additional file 1: Figure S3C). Finally, to prove that NTCP expression was directly induced by 1 μM Dexa via the glucocorticoid receptor (GR), we tested a known GR antagonist on D-UCMSCs, the anti-glucocorticoid RU486 which blocks the ligand-induced action of GR. Indeed, the addition of 1 μM RU486 with Dexa after 4 days of treatment significantly reduced NTCP mRNA expression in D-UCMSCs and fully inhibited the effect of Dexa as compared 1 μM Dexa without RU4846 (*p* < 0.001; *t* test) (Fig. 3e).

### NTCP protein expression in D-UCMSCs

**Fig. 4:**
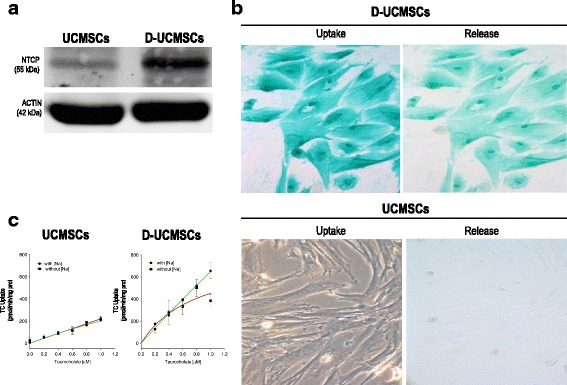
NTCP protein modulation in D-UCMSCs. **a** Western blot analysis of sodium taurocholate cotransporting peptide (*NTCP*) protein expression in differentiated umbilical cord stem cells (*D-UCMSCs*): an NTCP 55 kDa band was detected in D-UCMSCs (*n* = 3). Ninety micrograms of total protein samples were loaded per lane. NTCP was detected by anti-NTCP primary antibody (kind gift from Prof. Bruno Stieger). Actin was used as the loading control. **b** ICG functional uptake and release assay. D-UCMSCs acquire both the ability to internalize (*left panel*) and to release ICG (*right panel*). Pictures were taken at 5 h and at 5 days post-treatment, respectively (magnification 200×) (*n* = 3). In contrast, naive UCMSCs are not able to either internalise nor release ICG as compared to D-UCMSCs (*n* = 3). **c** Taurocholate (*TC*) uptake assay with and without sodium. Uptake was measured in UCMSCs (*n* = 2) and D-UCMSCs (*n* = 3) using increasing concentrations of TC. UCMSCs do not uptake TC efficiently with and without sodium (*left panel*). D-UCMSCs increase TC uptake function with or without sodium ((V_max_ = 1,460 *km* = 6.180). TC uptake under sodium conditions significantly increased at 1 μM (V_max_ = 10,791) as compared to the sodium-free condition (V_max_ = 821). Values are expressed as TC uptake (pmol/min/mg protein)

To confirm that NTCP mRNA expression can be effectively correlated to an enhanced protein expression, we used Western blot assay. In D-UCMSCs, high expression of a glycosylated NTCP could be demonstrated (visualized by Western blot as a prominent 55 kDa band; Fig. 4a) as compared to naive UCMSCs. Furthermore, to functionally characterize NTCP transporter activity in D-UCMSCs, ICG clearance assay was applied before and after hepatogenic differentiation. ICG is a compound that mimics organic anions, such as bile acid and bilirubin, and has been shown to be transported by NTCP [17]. As depicted in Fig. 4b, we could observe that ICG uptake was specific for D-UCMSCs (5 h post-treatment) that were also able to release it after 5 days. These results suggested that D-UCMSCs can take up ICG via NTCP-mediated active transport, even though we cannot exclude the involvement of additional transporters such as OATPs isoform 3A1 [17]. Finally, to confirm NTCP-specific sodium-dependent transport activity, we performed taurocholate (TC) uptake assays with or without sodium for both UCMSCs and D-UCMSCs (Fig. 4c). In line with an overall low NTCP expression in UCMSCs, the TC uptake capacity of UCMSCs was not significantly changed in sodium versus sodium-free conditions (Vmax = 1.460; *km* = 6.180 μM). By contrast, in D-UCMSCs overall TC uptake rates were markedly increased under conditions in which sodium ions were available (Vmax = 10.791; *k*_*m*_ = 15.65; R_2_ = 0.96) as compared to sodium-free conditions (V_max_ = 821; *k*_*m*_ = 0.817; R_2_ = 0.807), particularly at 1 μM.

### Inhibition of HBV entry in D-UCMSCs by taurocholate

**Fig. 5:**
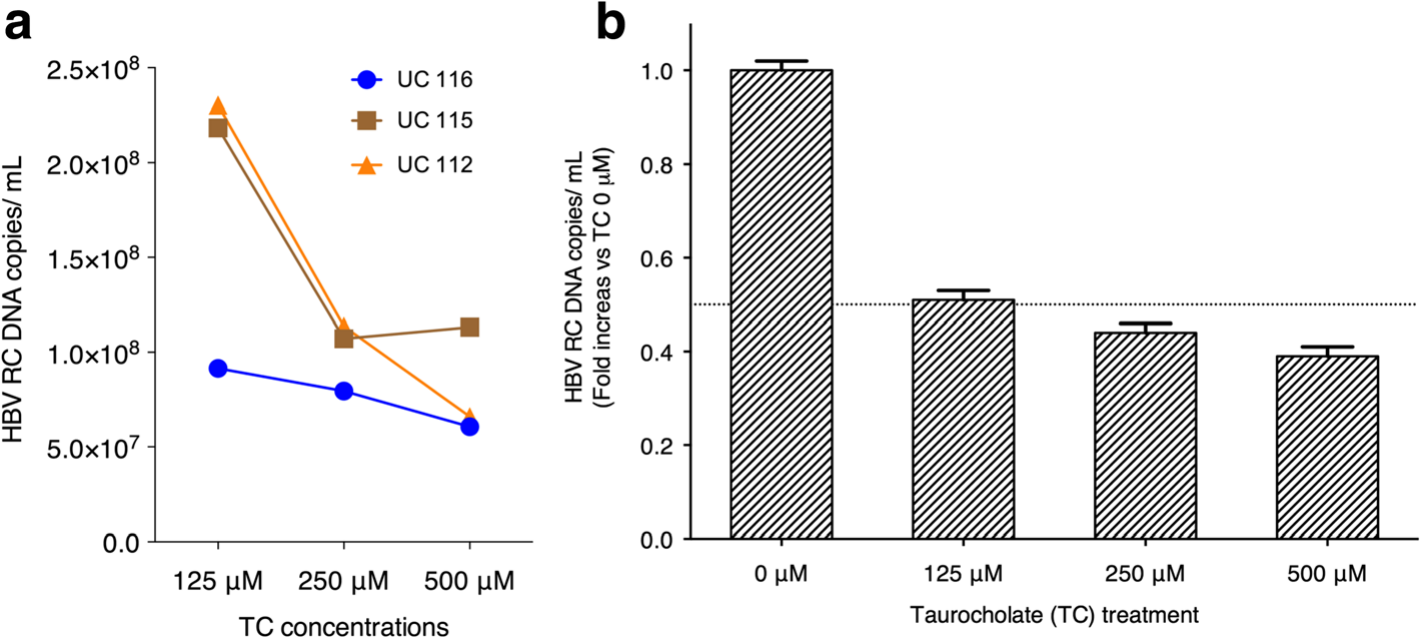
Inhibition of HBV entry in D-UCMSCs. **a** Inhibition of viral entry by taurocholate (*TC*) treatment: infected D-UCMSCs were treated 2 h prior to and during infection with different concentrations of TC. Supernatant was collected at day 4. Secreted HBV relaxed circular (*RC*) DNA virions were quantified by absolute qPCR assay. Values are expressed as absolute copys numbers of HBV RC DNA infectious virions at different TC concentrations and produced by three different donors. **b** Infected D-UCMSC TC treatment relative to non-treated: treated cells as compared to non-treated cells present a substantial decrease in HBV production when increasing TC concentrations are used (*n* = 3). Absolute quantification of HBV RC DNA positive virions is expressed as fold increase versus cells 24 h after primary infection

To prove that D-UCMSC infection by HBV was mediated by NTCP, we treated D-UCMSCs with TC, a specific NTCP inhibitor [7], before and during the complete time span of the infection experiments. To inhibit the HBV entry process we used the same TC concentrations as previously documented in other studies to inhibit HBV entry [7]. To assess the degree of infection viral particles, supernatants were collected at day 4 from D-UCMSC conditioned medium and qPCR absolute quantification of HBV RC DNA virions was performed. In Fig. 5a we show that in all TC concentrations tested the total viral production over 4 days post-infection was significantly reduced while 500 μM TC most profoundly influenced the infection process in D-UCMSCs. To evaluate the effect of viral inhibition in D-UCMSCs, we compared viral production between D-UCMSCs treated with TC and non-treated controls at day 4 post-infection. In Fig. 5b, we show that 125 μM TC treatment reduced viral production in D-UCMSCs by 50% (0.5 ± 0.2 SD) as compared to untreated cells. In addition, infected D-UCMSCs treated with another physiological NTCP substrate taurochenodeoxycholate (TCDC) proved that TCDC treatment at all concentrations tested (0.2 μM, 0.75 μM, 1 μM, 1.25 μM) also inhibited viral production at day 4 post-infection as compared to non-treated D-UCMSCs (*n* = 2) (Additional file 1: Figure S5B).

### Hepatocytic gene modulation in HBV-infected D-UCMSCs

**Fig. 6:**
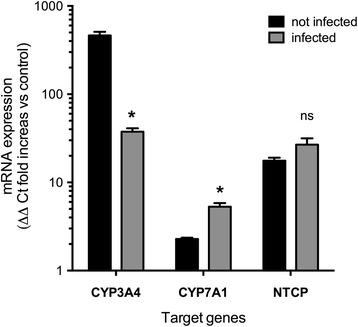
HBV gene modulation in infected D-UCMSCs. Gene expression analysis between HBV infected and non-infected cells shows that sodium taurocholate cotransporting peptide (*NTCP*) expression was not significantly modulated by HBV (*p* = not significant by multiple *t* test; *n* = 3). In contrast, HBV infection significantly decreases CYP3A4 expression (462.6 ± 47.4 SEM; *p* = 0.0008) and increases CYP7A1 expression (2.2 ± 0.081 SEM; *p* = 0.005). Values are expressed as relative quantity compared to UCMSCs that were not infected

Finally, to study HBV-cell interactions, we compared D-UCMSCs that had been infected with HBV to non-infected cells. As depicted in Fig. 6, HBV infection significantly decreased the CYP3A4 mRNA expression (37 ± 0.5 SEM) as compared to non-infected (462 ± 47.5 SEM) levels, whereas CYP7A1 mRNA expression increased (5.29 ± 0.5 SEM) as compared to non-infected (2.28 ± 0.5 SEM) levels. With respect to NTCP, a tendency of an increased expression was observed as compared to non-infected cells.

## Discussion

NTCP is the major uptake transporter for bile salts in hepatocytes [18]. Recently, NTCP has been discovered to be the first functional HBV receptor to enable HBV entry in NTCP-expressing permissive cells. The present study characterizes the expression of NTCP in D-UCMSCs as a marker of viral entry and as a new indicator of stem cell hepatogenic differentiation quality [18]. None of the previous studies conducted on other MSCs has ever evaluated NTCP expression upon an in-vitro hepatogenic differentiation protocol. Indeed, in D-UCMSCs, NTCP mRNA and protein expression was upregulated and correlated with an enhancement of TC uptake activity as compared to naive UCMSCs (Figs. 3 and 4). We also demonstrate that NTCP inhibitors decreased HBV entry in D-UCMSCs as compared to non-treated cells, suggesting a competitive binding for NTCP (Fig. 5). Our data prove that D-UCMSCs express functional NTCP and that in this in-vitro infection model of HBV entry depends on its functionality. Isolated hepatocytes, in comparison to D-UCMSCs, constitutively express NTCP, but rapidly lose NTCP activity unless complex culture conditions are optimized [15]. As such, a valid alternative to PHH culture is the use of D-UCMSCs to specifically modulate NTCP expression by using dexamethasone and avoiding complex culture conditions that might interfere with viral entry.

HBV entry is a key process for developing a productive infection. Hence, it represents an attractive molecular target for the design of new antiviral strategies to block viral re-infection, a still poorly understood process [9]. The extremely restricted tropism of HBV and the limited in-vitro models available have hampered its study. Glycosaminoglycans (GAGs), in particular heparan sulfate proteoglycans (HSPGs), have been demonstrated to be essential for the initial HBV attachment process, but these are not sufficient for infection simply because these proteoglycans are not specific for susceptible cells [19, 20]. Indeed, the findings of Schulze et al. were recently confirmed by the identification of a subclass of HSPGs called glypican 5 (GPC-5) [21]. In contrast, HBV binding and uptake depend on the expression and function of the NTCP transporter, strongly suggesting that this membrane-bound protein is the cell host determinant for HBV tropism [7, 8].

Nevertheless, other molecular determinants might be required for a productive and efficient infection since it has been reported that 100% infection efficiency is not reached even in transfected NTCP-overexpressing cells [8, 22, 23]. Therefore, the optimization of new in-vitro infection models is needed to investigate the detailed mechanisms of the HBV viral entry process and its modulation in greater depth. At present, HBV in-vitro infection models are limited and are all suboptimal. Several common disadvantages of currently available cell systems are a low replication efficiency, high MOIs needed, and the use of molecular adjuvants, such as DMSO and/or PEG, to enhance infection [5]. Therefore, D-UCMSCs are a suitable tool for the study of HBV early events, although it should be noted that high MOIs are needed to produce an efficient infection. Nevertheless, molecular adjuvants are avoided in D-UCMSC infection studies which position this cell model close to a more physiological condition [12]. Our data also show that increased NTCP expression occurs during the last step of the hepatogenic differentiation via Dexa. This effect is mediated via GR activation and suggests an indirect modulation of HBV infection through this glucocorticoid receptor [24]. Indeed, RXR, a transcription factor which binds to the NTCP promoter, was recently proven to enhance HBV infection by upregulating NTCP expression [25].

UCMSCs are easily and reproducibly isolated from a regularly available source and maintain their features even after cryopreservation. Upon in vitro hepatogenic differentiation, D-UCMSCs express NTCP and enhance susceptibility to HBV. Here, we describe D-UCMSC viral kinetics and observe that the HBV infection process is rapid and robust, with no cytopathic effect over 4 days post-infection. To monitor the HBV life cycle, we analyzed D-UCMSC supernatant for HBV RC DNA virions by qPCR analysis. Indeed, the detection of mature virions represents indirect proof that pgRNA packaging and nucleocapsid formation are completed without error by D-UCMSCs. Such results were validated after cccDNA detection using ddPCR assay and by demonstrating the expression of pgRNA viral transcripts.

We monitored cccDNA biogenesis every 24 h in D-UCMSCs over 4 days and found that the number of cccDNA copies detected per cell was between 0.02 and 0.06, which is higher than that previously reported at 24 h post-infection (<0.02 copies/cell) [12]. However, it is still less than the cccDNA detected in HepaRG (0.5 copies/cell), PHH (1 to 2 copies/cell), and HepG2-NTCP (1 to 5 copies/cell) infection models [26]. The low cccDNA copy number detected in D-UCMSCs might be influenced by the non-use of adjuvants during the infection step. Nevertheless, D-UCMSCs are a valid model to study cccDNA formation and also a tool for targeting the HBV genome, in which cccDNA biogenesis can be monitored by quantitative ddPCR analysis.

To determine the critical involvement of functional NTCP in HBV-induced infection of D-UCMSCs, we pre-exposed the differentiated cells to TC and, as expected, a marked decrease in HBV infection could be achieved, clearly suggesting a competition between HBV binding and TC for the available TC binding sites [7, 27]. Our findings are in line with previous reports demonstrating that the NTCP substrates TC, TDC, and TCDC reduce infection by interfering with the HBV viral entry process [7]. HBV can modulate several cellular host pathways. Previous reports have shown that cytochrome P450 isoforms (CYPs; liver-specific enzymes) are modulated by HBV [28]. In particular, CYP7A1 expression was found to increase in vivo post-HBV infection [29]. In line with these findings, we could demonstrate for the first time that HBV infection increases CYP7A1 mRNA expression in D-UCMSCs, while CYP3A4 expression was decreased. No significant modulation of NTCP expression was noticed post-HBV infection in D-UCMSCs (Fig. 6). These experiments are the first attempt to study hepatocytic gene modulation in infected D-UCMSCs and demonstrate that virus-host interaction studies might be useful to investigate HBV liver-induced diseases in a controlled microenvironment.

## Conclusions

The present study proposes D-UCMSCs as a suitable HBV infection system to study viral entry and the life cycle. D-UCMSC HBV infection efficiency can be improved by increasing the quality of differentiation and by modulating NTCP expression, for instance as shown here after the activation of GR by Dexa. A recent study on iPSC-derived hepatocytes has used NTCP as a marker to obtain a homogenous hepatocyte-like population expressing NTCP, but these authors did not prove infection with HBV [30]. A similar enrichment approach might be suitable to differentiated D-UCMSCs in order to augment the infection rate. Taken together, our results suggest that the HBV life cycle depends on the expression and functionality of NTCP in D-UCMSCs, which enables HBV entry and modifies cellular gene expression patterns.

## Additional file

Additional file 1: Figure S1. Naive and differentiated (three-step differentiation validation) MSC characterization. **Figure S2.** HBV infection validation. **Figure S3.** NTCP mRNA expression. **Figure S4.** NTCP protein expression. **Figure S5.** HBV-NTCP entry inhibition. **Table S1.** Primary antibodies used for MSC characterization. (ZIP 1866 kb)

## Abbreviations

bFGF: Basic fibroblast growth factor
BSA: Bovine serum albumin
cccDNA: Covalently closed circular DNA
CYP3A4: Cytochrome P450 3A4
dd: Digital droplet
Dexa: Dexamethasone
DMSO: Dimethyl sulfoxide
D-UCMSC: Differentiated umbilical cord mesenchymal stem cell
GR: Glucocorticoid receptor
HBV: Hepatitis B virus
HGF: Hepatocyte growth factor
hNTCP: Human sodium-taurocholate cotransporting peptide
HSPG: Heparan sulphate proteoglycan
ICG: Indocyanine green
IMDM: Iscove’s modified Dulbecco’s medium
ITS: insulin-selenium-transferrin
MOI: Multiplicity of infection
NTCP: Sodium-taurocholate cotransporting peptide
PBS: Phosphate-buffered saline
pgRNA: Pregenomic RNA
PHH: Primary human hepatocyte
P/S: Penicillin/streptomycin
qPCR: Real-time quantitative polymerase chain reaction
RC: Relaxed circular
TC: Taurocholate
TCDC: Taurochenodeoxycholate
UCMSC: Umbilical cord mesenchymal stem cell
