# Supplementary material for: Upregulation of sodium taurocholate cotransporter polypeptide during hepatogenic differentiation of umbilical cord matrix mesenchymal stem cells facilitates hepatitis B entry
Source: Stem Cell Res Ther. 2017 Sep 29;8:204. doi: 10.1186/s13287-017-0656-5 (PMC5622580; doi:10.1186/s13287-017-0656-5)
Supplement: Additional file 1: Figure S1. — Naive and differentiated (three-step differentiation validation) MSC characterization. Figure S2. HBV infection validation. Figure S3. NTCP mRNA expression. Figure S4. NTCP protein expression. Figure S5. HBV-NTCP entry inhibition. Table S1. Primary antibodies used for MSC characterization. (ZIP 1866 kb) [file 13287_2017_656_MOESM1_ESM.zip › Sargiacomo et al_supplementary figures_R3_final.pptx]

## Slide 1
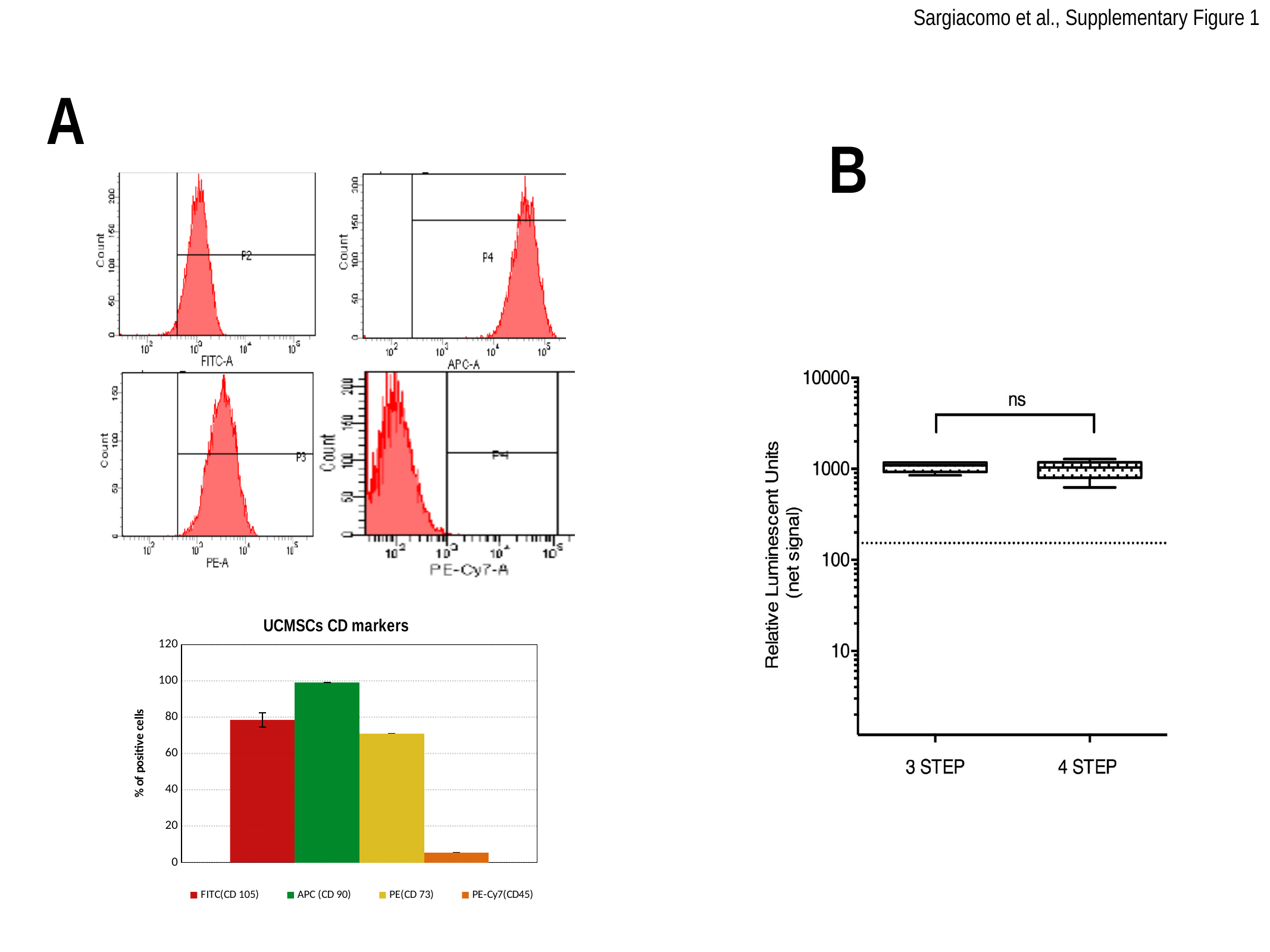

Sargiacomo et al., Supplementary Figure 1
A
B
### Chart: UCMSCs CD markers
| Category | FITC(CD 105) | APC (CD 90) | PE(CD 73) | PE-Cy7(CD45) |
|---|---|---|---|---|
| CD 90 | 78.5 | 99.116667 | 71.05 | 5.55 |

## Slide 2
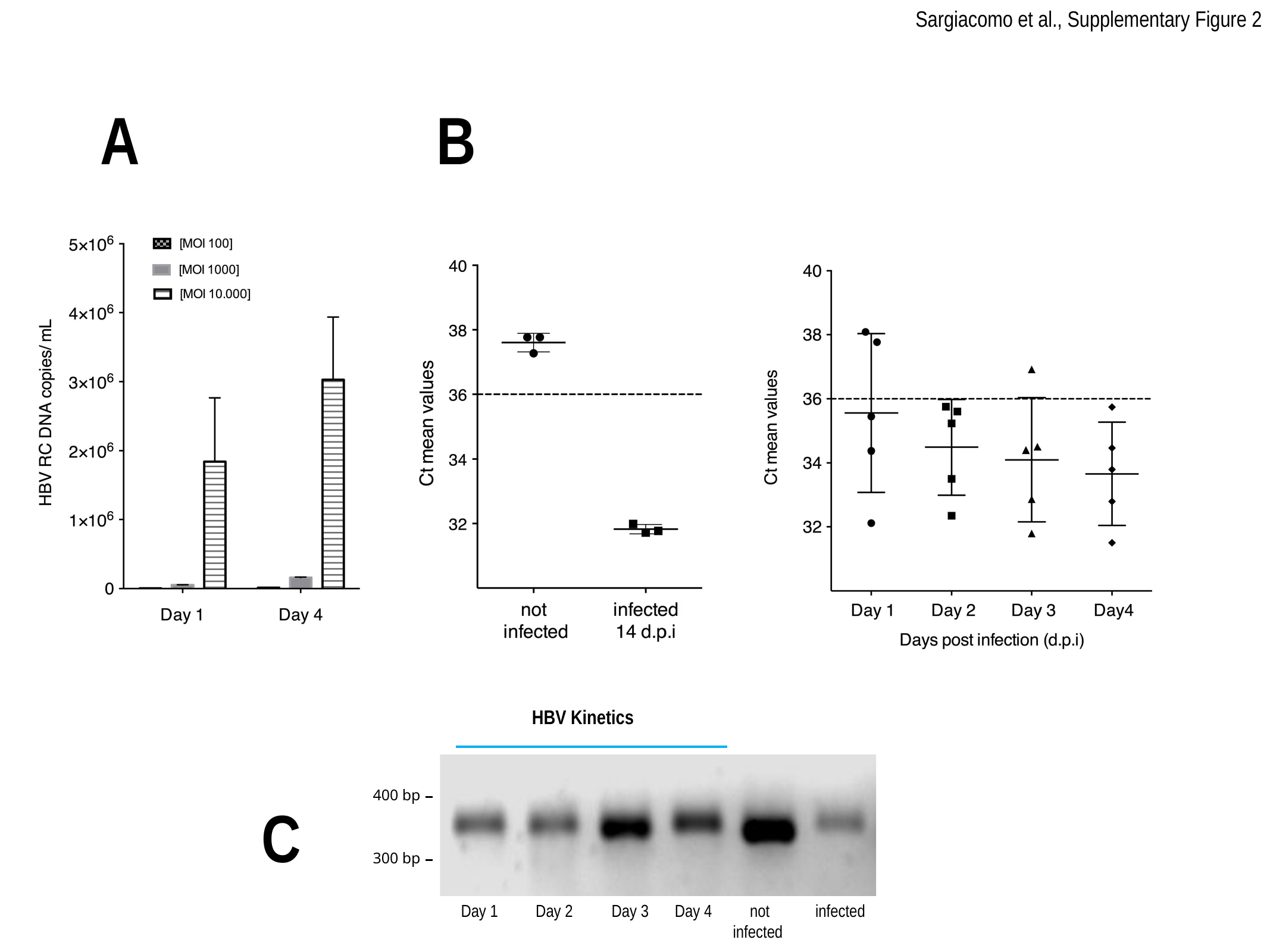

Sargiacomo et al., Supplementary Figure 2
A
B
HBV Kinetics
400 bp
C
300 bp
Day 1
Day 2
Day 3
Day 4
not
infected
infected

## Slide 3
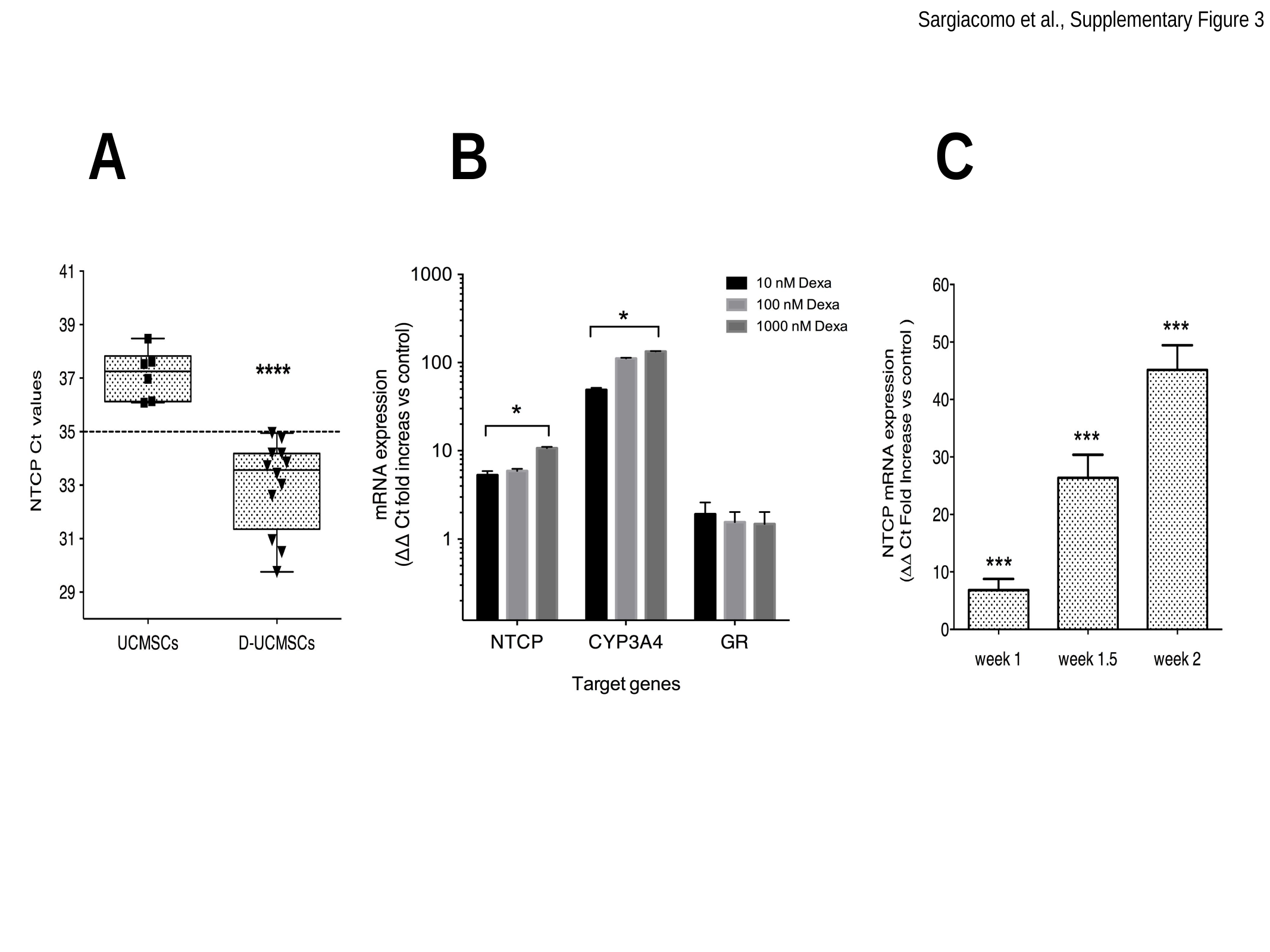

Sargiacomo et al., Supplementary Figure 3
B
A
C

## Slide 4
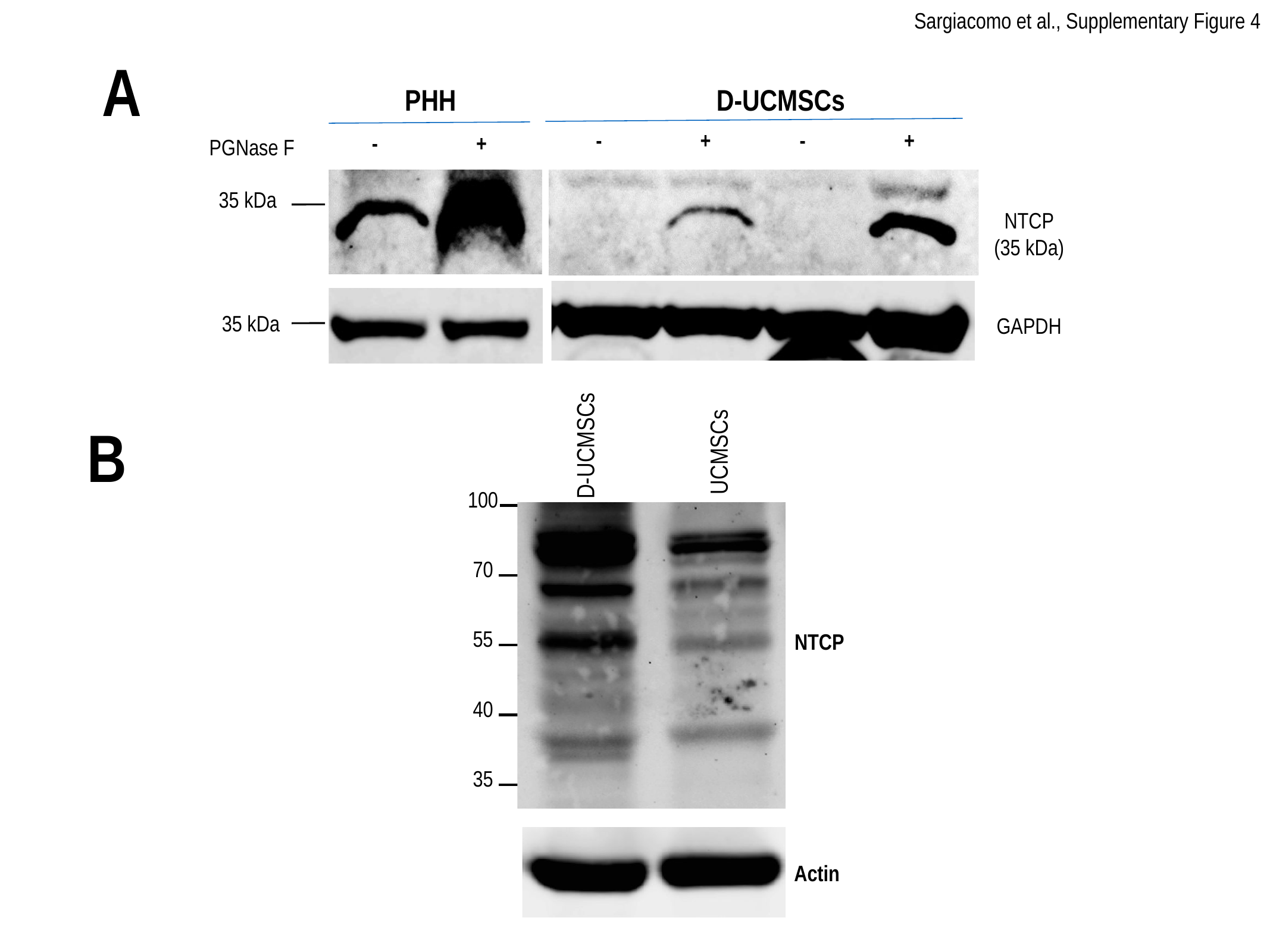

Sargiacomo et al., Supplementary Figure 4
A
PHH
D-UCMSCs
- 	 +
- 	 +
- 	 +
35 kDa
NTCP
(35 kDa)
35 kDa
GAPDH
PGNase F
B
D-UCMSCs
 UCMSCs
100
70
NTCP
55
40
35
Actin

## Slide 5
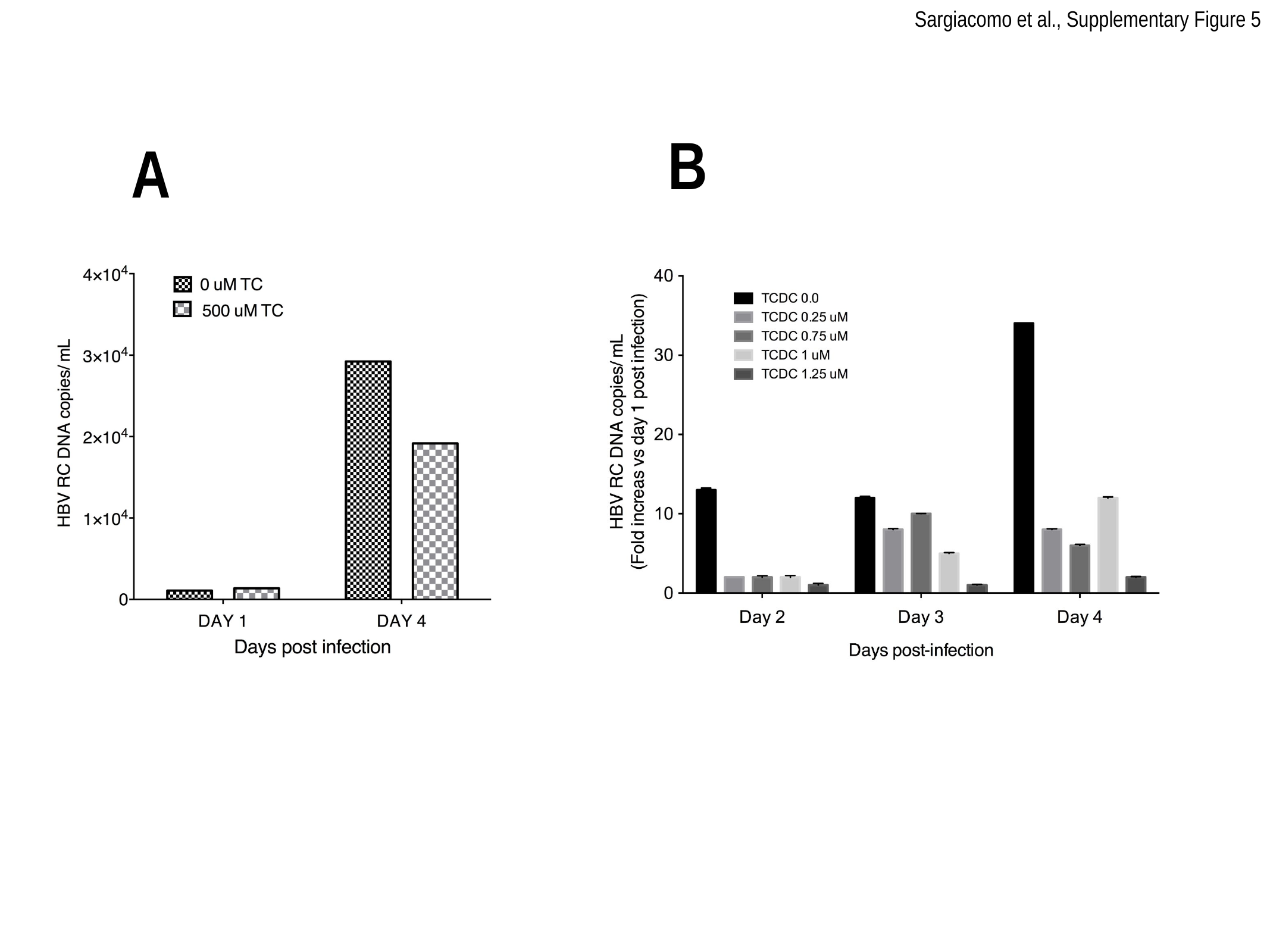

Sargiacomo et al., Supplementary Figure 5
B
A
