# Supplementary material for: Upregulation of sodium taurocholate cotransporter polypeptide during hepatogenic differentiation of umbilical cord matrix mesenchymal stem cells facilitates hepatitis B entry
Source: Stem Cell Res Ther. 2017 Sep 29;8:204. doi: 10.1186/s13287-017-0656-5 (PMC5622580; doi:10.1186/s13287-017-0656-5)
Supplement: Additional file 1: Figure S1. — Naive and differentiated (three-step differentiation validation) MSC characterization. Figure S2. HBV infection validation. Figure S3. NTCP mRNA expression. Figure S4. NTCP protein expression. Figure S5. HBV-NTCP entry inhibition. Table S1. Primary antibodies used for MSC characterization. (ZIP 1866 kb) [file 13287_2017_656_MOESM1_ESM.zip › Sargiacomo et al_SUPPLEMENTARY MATERIAL_R3_Final.docx]

Supplementary Material

##### ***Supplementary Figure 1 (S1): naïve and differentiated (3 step differentiation validation) MSCs characterization***

1. UCMSCs characterization by flow cytometry: Freshly isolated UCMSCs were characterized prior differentiation for their MSCs markers. UCMSCs expressed CD90 (99%), CD105 (78.5%) and CD73 (71%) markers; whereas, they were negative for CD45 (5.5%) hematopoietic marker. Values are expressed as percentage of positive cells (*p*<0.05) (n=6).
2. CYP3A4 activity assay after 3 Steps and 4 Steps hepatogenic differentiation protocols. No significant differences in CYP3A4 activity levels were noticed between both protocols (*p*=ns) (Mann-Whitney U test). UCMSCs CYP3A4 baseline activity mean value (dash line). Values are expressed as relative light units (RLU) net signal.

##### ***Supplementary Figure 2 (S2): HBV infection validation***

1. Infected D-UCMSCs supernatant was analysed at day 1 and day 4 post-infection. Detectable amounts of viral production were produced in D-UCMSCs by using MOIs of 1,000 and 10,000. MOIs of 2,500 were used as a standard infection inoculum in the study. Values are expressed as HBV RC DNA absolute values per mL of infected cell culture medium.
2. HBV cccDNA assay validation: At fourteen days post-infection, DNA from infected D-UCMSCs was extracted and measured by conventional qPCR using cccDNA TaqMan assay. cccDNA was detectable in infected D-UCMSCs with a mean Ct value of 32 as compared to 37 in non-infected cells (left graph). HBV cccDNA kinetic studies proved that infection is rapid and sustained over time (right graph). Threshold was arbitrary established at 36 (dash line). Values are expressed as Ct values in triplicate (inter-assay) (n=1).
3. cccDNA TaqMan assay specificity was tested by running the qPCR post-PCR production on agarose gel (1.5%). cccDNA assay produced a single specific band of 350 bp in all infected D-UCMSCs whereas a background signal was detectable in non-infected D-UCMSCs.

##### ***Supplementary Figure 3 (S3): NTCP mRNA expression***

1. NTCP Ct values were compared between UCMSCs (Median=37.3 ± 0.9 SD (n=6)) and D-UCMSCs (Median=33.6 ± 1.7 SD (n=12) (*p*<0.0001; Unpaired t test; Two tail). NTCP expression threshold was arbitrarily chosen as 35 (black line). 250 ng of cDNA was used for each qPCR reaction.
2. Dexa modulates D-UCMSCs gene expression: Increasing concentrations of Dexa (10 nM, 100 nM, 1,000 nM) (n=2) were tested on D-UCMSCs at the last Step of hepatogenic differentiation. qPCR analysis shows that both NTCP mRNA and CYP34 mRNA expression significantly increased using 1,000 nM as compared to 10 nM (*p*<0.01), whereas GR mRNA expression was not modulated by Dexa (*p*=ns). Values are expressed as mRNA ∆∆Ct fold increase and compared to UCMSCs at Step 2 of hepatogenic differentiation.
3. Dexa 10^-6^ M induces NTCP mRNA expression in a time-dependent manner: Prolonged hepatogenic differentiation increases NTCP mRNA expression in a time-dependent manner as compared to the standard D-UCMSCs protocol. Values are expressed as NTCP ∆∆Ct fold increase as compared to day 0 (end of differentiation). In each bar, one single donor is expressed by NTCP fold increase values (n=3). Two replicates using three donors one for each time point.

***Supplementary Figure 4 (S4): NTCP protein expression***

**A.** NTCP deglycosylation assay. PHH and D-UCMSCs protein extracts were treated with PGNase F and analyzed by SDS-PAGE. As a result, NTCP 38 kDa non-glycosylated band was enhanced in treated conditions as compared to non-treated D-UCMSCs.

**B.** Full SDS-PAGE gel image of NTCP western blot analysis in naïve and D-UCMSCs (Figure 4A). Ninety micrograms of total protein samples were loaded per lane. NTCP was detected by an anti-NTCP primary antibody (kind gift from Prof. Bruno Stieger). Actin was used as loading control

***Supplementary Figure 5 (S5): HBV-NTCP entry inhibition***

1. Inhibition of D-UCMSCs infection by HBV after co-incubation with 500 µM TC. Supernatant from TC treated and untreated D-UCMSC cultures, was recovered at day 1 and day 4 post-HBV infection. Secreted HBV RC DNA virions were analyzed after absolute quantification and expressed as HBV RC DNA copies/mL.
2. Infected D-UCMSCs were treated with increasing amounts of TCDC (0 µM, 0.2 µM, 0.75 µM, 1 µM, 1.25 µM) over four days. Viral production by D-UCMSCs significantly decreased with all TCDC concentrations as compared to non-treated D-UCMSCs (n=2).

##### ***Supplementary Table 1: Primary antibodies used for MSCs characterization***

| **Antibody** | **Supplier** | **Reference** | **Species** | **Concentration used** |
| --- | --- | --- | --- | --- |
| CD90-APC | BD Bioscience | 559869 | Mouse anti-human | 1/10 |
| CD73-PE | BD Bioscience | 550257 | Mouse anti-human | 1/10 |
| CD105-FITC | BD Bioscience | 561443 | Mouse anti-human | 1/10 |
| CD45-Cy7 | BD Bioscience | 561868 | Rat anti-mouse | 1/10 |
